# Gait variability and local dynamic stability are exacerbated by age and visual cues

**DOI:** 10.7717/peerj.21157

**Published:** 2026-05-05

**Authors:** Jonaz Moreno Jaramillo, Paul McDonnell, Adam Grimmitt, Wouter Hoogkamer, Douglas N. Martini

**Affiliations:** Department of Kinesiology, University of Massachusetts at Amherst, Amherst, MA, United States of America

**Keywords:** Gait variability, Local dynamic stability, Short-term Lyapunov exponent, Age, Old adults, Young adults, Visual information, Overground, Treadmill, Visual cues

## Abstract

**Background:**

Gait variability increases and dynamic gait stability decreases with age, which may lead to an increased risk of falls. Additionally, the need to process visual cues can exacerbate changes in gait variability and dynamic gait stability in older adults. The purpose of this study was to compare gait variability and dynamic gait stability in young and older adults walking overground and on a treadmill with and without visual cues. We hypothesized that gait variability would increase and dynamic gait stability would decrease during the visually cued gait. We also hypothesized that older adults would have higher variability and lower stability than younger adults across all walking conditions.

**Methods:**

Participants walked for 3 minutes overground and on a treadmill, with and without visual cues, at their self-selected overground walking speed. The visual cues were projected onto the treadmill and matched the participant’s self-selected speed, step length, and width. We quantified gait variability as the coefficient of variation for stride time and stride length, and dynamic gait stability with the short-term Lyapunov exponent.

**Results:**

Both groups had the highest stride time variability during the visual cue condition. Older adults demonstrated higher gait variability and lower dynamic gait stability than younger adults during the treadmill conditions with and without visual cues, but not during overground gait.

**Conclusions:**

Our study indicates that age increases gait variability and decreases dynamic gait stability, and this effect is exacerbated when walking becomes more challenging due to the presence of visual cues. The observed changes in gait variability and dynamic gait stability may be due to older adults requiring more cognitive capacity.

## Introduction

Advanced age is associated with higher gait variability and lower dynamic gait stability, which are associated with increased fall risk in community environments (Hausdorff, Rios & Edelberg, 2001; Brach et al., 2007; Ihlen et al., 2016). Consistent evidence indicates that falls can cause serious injuries, reduce the quality of life, and incur high healthcare costs for older adults (Robinovitch et al., 2013; Woolrych et al., 2015). Quantifying changes in gait variability and stability in the laboratory can inform why older adults experience increased fall risk compared to younger popluations (Lockhart & Liu, 2008; Toebes et al., 2012; Bizovska et al., 2018a). However, assessing variability and stability during simple (*i.e.,* unperturbed) and controlled gait conditions (*e.g.*, treadmill walking) may not be representative of gait behavior in more complicated environments.

Gait variability changes across conditions and with age (Bizovska et al., 2018b; Schmitt et al., 2021; Hybart & Ferris, 2023) and appears to be associated with an increased risk of falls (Hausdorff et al., 1997; Hausdorff, Rios & Edelberg, 2001). Stride time and stride length variability are typically reported for overground and treadmill walking in a laboratory environment and when walking outside the laboratory using inertial measurement units (IMUs) (Washabaugh et al., 2017; Tamburini et al., 2018; Renggli et al., 2020; Schmitt et al., 2021; Matikainen-Tervola et al., 2024). For example, older adults with a history of falls have a higher stride time variability during overground walking (Hausdorff et al., 1997; Hausdorff, Rios & Edelberg, 2001). Importantly, prospective studies have reported that older adults with higher stride length variability are at increased risk of falls (Maki, 1997; Callisaya et al., 2011). More recently, Doi et al. (2020) observed that higher stride length variability during walking is associated with higher disability certifications, putting this population at higher risk of falling. It has been demonstrated that stride time and stride length variability can be different between overground and treadmill walking, although results across studies are inconsistent. For stride time variability, Bizovska et al. (2018b) reported that both young and older women exhibited lower stride time variability during treadmill walking than during overground walking (23-meter bout indoors), with no significant differences between the two groups. On the other hand, Schmitt et al. (2021) observed no differences between treadmill and overground walking (8-meter bout indoors) but reported higher variability in older adults than in young adults across conditions. For stride length variability, Schmitt et al. (2021) also did not observe differences between treadmill and overground walking in both young and older adults, whereas Hybart & Ferris (2023) found higher stride length variability when young adults walked around a university campus for ∼2 km compared to treadmill walking. These findings suggest that gait variability is influenced by task context (Schmitt et al., 2021), personal factors such as physical and cognitive capacity, and/or situational factors such as human behavior and the environment (Rispens et al., 2016). On the cognitive side, increased gait variability outside the laboratory may be due to the fact that older adults require more attention to walk in complex conditions (Reuter-Lorenz & Cappell, 2008), suggesting reduced gait automaticity and a possible mechanism for increasing gait variability (Decker et al., 2016).

Despite the changes in gait variability in older adults, more variability by itself is not always worse (Van Emmerik & Van Wegen, 2000; van Emmerik & van Wegen, 2002; Stergiou, Harbourne & Cavanaugh, 2006). Therefore, quantifying dynamic gait stability, in addition to variability measures, can provide deeper insights into the gait performance of older adults. Dynamic gait stability is typically quantified by calculating the short-term Lyapunov exponent (Dingwell & Cusumano, 2000; Dingwell et al., 2001; Bruijn et al., 2013), commonly referred to as the Local Divergence Exponent (LDE). Young and older adults walk with a higher LDE (*i.e.,* less stable gait) during overground walking than treadmill walking (Dingwell et al., 2001; Buzzi et al., 2003; Terrier & Dériaz, 2011; Bizovska et al., 2018b; Amirpourabasi et al., 2022). Further, young adults walk with higher LDE in challenging walking environments induced by visual flow perturbations (McAndrew, Wilken & Dingwell, 2011; Wuehr et al., 2013; Francis et al., 2015). Importantly, previous studies suggest that a higher LDE value may indicate a higher risk of falls in older adults (Lockhart & Liu, 2008; Toebes et al., 2012; Rispens et al., 2015; Ihlen et al., 2016; Bizovska et al., 2018a). Measuring dynamic gait stability under conditions that are different from normal treadmill walking can provide a more comprehensive assessment of gait performance. As such, we applied a visual cue treadmill walking paradigm to assess whether added visual flow information affects gait performance (Cates & Gordon, 2022; Cates & Gordon, 2023). We projected illuminated rectangles that moved at the same speed as the treadmill belt, allowing participants to step on them while walking.

Both gait variability and stability change in response to age, visual flow information (*i.e.,* cues), and walking environment (*i.e.,* overground *versus* treadmill) (McAndrew, Wilken & Dingwell, 2011; Wuehr et al., 2013; Francis et al., 2015). However, limited evidence across these factors under matched experimental conditions inhibits our ability to assert strong conclusions. We aimed to quantify gait variability and dynamic gait stability during overground and treadmill gait conditions, with and without visual cues in young and older adults. Based on previous reports of gait variability and dynamic gait stability (Dingwell et al., 2001; Bizovska et al., 2018b; Schmitt et al., 2021; Hybart & Ferris, 2023), we hypothesized that stride time variability would decrease, stride length variability would increase, and that dynamic gait stability would increase (*i.e.,* lower LDE) during treadmill walking compared to overground walking. We also hypothesized that stride time and length variability would increase and dynamic gait stability would decrease (*i.e.,* higher LDE) during visual cue treadmill gait compared to treadmill walking without visual cues in both young and older adults. Finally, we hypothesized that older adults would walk with higher stride time and stride length variability and lower dynamic gait stability than young adults across all three gait conditions.

## Materials & Methods

### Participants

Twenty-five young adults (mean [SD] = 22 [4] years old; females = 15) and twenty-five older adults (mean [SD] = 70 [4] years old; females = 12) participated in this study. Written informed consent was obtained from each participant before the study. All procedures were approved by the Institutional Review Board (protocol #3501) at the University of Massachusetts Amherst. The inclusion criteria were that young adults were between 18 and 29 years old, while older adults were 65 years old or older. The exclusion criteria included having a chronic musculoskeletal or neurological injury or disease, undergoing surgery, or experiencing an injury that would affect gait within the past year; having a cardiovascular disease or arrhythmia, uncorrected vision impairment; and being unable to understand written and spoken English.

### Protocol

The older adult group completed the Montreal Cognitive Assessment (MoCA) (mean [SD] = 26 [1.90], min = 23, max = 30; Freitas et al., 2012) and the Physical Activity Scale for Elderly (PASE) questionnaire (mean [SD] = 183 [64], min = 86, max = 371) after informed consent was obtained and before performing any gait conditions. The MoCA assessment was used for screening purposes, to account for any cognitive impairment in the older adult group. Additionally, the PASE assessment was used to quantify physical activity levels. A PASE score of ≤ 67 may indicate physical inactivity (Ayvat, Doğan & Ayvat, 2025).

All Participants wore six OPAL v2 IMUs (APDM, a Clario Company, Portland, OR, USA) sampling at 128 Hz: one on each foot, each wrist, the sternum (xyphoid process), and the lumbar spine (L5). IMUs were wirelessly synchronized *via* the Mobility Lab™ v2 software (APDM, a Clario Company, Portland, OR, USA). Stride time and length variability for both the left and right legs were computed by Mobility Lab™ as the standard deviation across all conditions. Participants completed three walking conditions, each lasting 3 min. First, the participants walked overground at their preferred walking speed along a 17-meter walkway marked by cones at either end, which served as turning points. The second and third conditions were performed on a dual-belt treadmill (Bertec, Colombus, OH, USA). In the second condition, the participants walked on the treadmill at their preferred walking speed, which was obtained from the overground condition. Participants without treadmill walking experience had a familiarization period starting at a lower speed than their preferred walking speed and ramping up by 0.5 meters/second every 30 s until their preferred speed was reached. For the third condition, visually cued gait, we used a short-throw projector to project illuminated rectangles onto the treadmill, which moved at the same speed as the belt. Participants were asked to step on the projected visual cues. Spacing and speed of the visual cues were pre-programmed in a custom Python script (Python 3.12.2). The spacing of the projections was based on the participant’s habitual step length and width during treadmill walking. The speed of the projections was based on the treadmill speed, which matched the participant’s preferred overground gait speed. Step length and width were calculated from the foot kinematic data obtained during the treadmill condition, using three retroreflective markers on the heel, the 5th metatarsal, and the 2nd phalanx. We recorded and processed the kinematic data using eight Miqus cameras (Qualisys AB, Gothenburg, Sweden) and a custom MATLAB script (MathWorks Inc., Natick, MA, USA). We scaled each stepping target to the participant’s foot size, based on the shoe size they reported.

### Data analysis

Our primary gait variability outcome measures were the percent coefficient of variation of stride time and of stride length. We used custom MATLAB scripts to calculate the mean stride length and time variability between the left and right legs from the Mobility Lab™ outputs. We then calculated the group percent coefficient of variation as $\%CV= \left( \frac{Standard~deviation}{mean} \right) \times 100$.

For dynamic gait stability, we used the raw acceleration data from IMUs at the lumbar spine to calculate the LDE using an open-source MATLAB script (Bruijn, 2023) and following previous work (Bruijn et al., 2013; Van Schooten et al., 2014; Mehdizadeh, 2019). Briefly, we time-normalized the data to 100 samples, implemented a time delay of 25 samples (1/4 of the gait cycle), and implemented a 9D space from the three acceleration vectors (XYZ) (Van Schooten et al., 2014). The LDE was calculated across all conditions using 11 bouts of eight strides. Previous work suggests that 15 bouts of eight strides are sufficient for a valid estimation of the LDE (Van Schooten et al., 2014). However, not all older adults covered 15 bouts of the 17-meter walkway during the overground condition, so we could not extract them. Instead, we used 11 bouts across all conditions to increase the total number of participants in our analysis. For participants who walked at least 15 lengths, we ran a paired *t*-test comparing 11 bouts *versus* 15 bouts of eight strides and found no difference in the two LDE values (*t* = −0.385; *p* = 0.701; Cohen’s *d* = 0.098).

### Statistics

All statistical analyses were conducted using JASP software (JASP 0.19.1; Amsterdam, The Netherlands). We analyzed all three outcome measures using a 2 × 3 mixed-effect ANOVA (age group × walking condition) for each outcome measure (*i.e.,* three separate mixed-effect ANOVA). A Greenhouse-Geisser correction was applied to the stride time and stride length variability, as sphericity was violated for these two variables. For the condition effect, we conducted a *post hoc* analysis with a Holm-Bonferroni adjustment to control familywise error and determine the significant pairwise comparisons. For the within condition group effect, we conducted a simple main effect analysis with a Holm-Bonferroni adjustment to determine which pairwise comparisons were significant. For the mixed-effect ANOVA, the partial eta squared (*η*^2^) was calculated and categorized as small (≥0.01), medium (≥0.06), and large (≥0.14) (Cohen, 1988). An *a prioriα* = 0.05 was used to establish statistical significance. A *post hoc* power analysis using G*Power 3.1.9.7 (Faul et al., 2007) indicated that our total sample size was adequate, with our lowest partial *η*^2^(0.086) resulting in a statistical power well above 80%.

## Results

### Stride time variability

**Figure 1:**
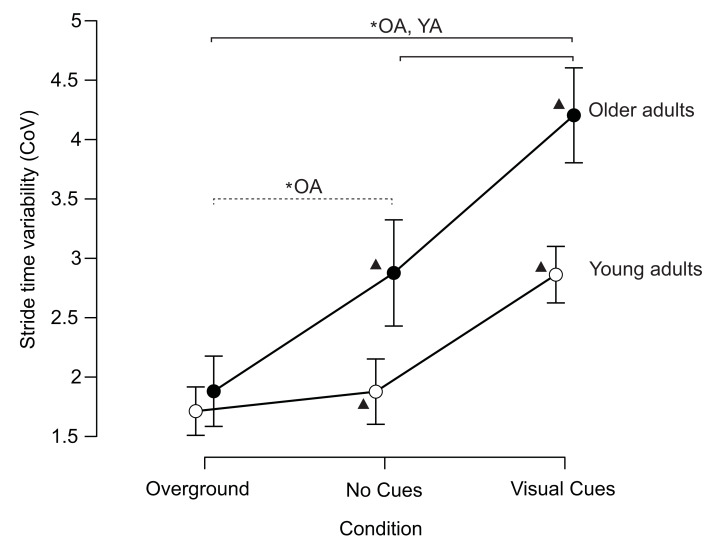
Older adults have a higher stride time variability than young adults when walking on a treadmill with and without visual cues. The overground stride time variability was similar between the two groups. CoV is the coefficient of variation. ▴ Indicates statistically significant differences between groups and for the same condition. The solid line indicates statistically significant differences for both groups across different conditions (OA, older adults; YA, young adults). The dashed line indicates statistically significant differences for the older adult group between the overground and treadmill without visual cues conditions. *: *p* < 0.05. The error bars indicate the 95% confidence interval.

Older adults walked with higher stride time variability than young adults (significant main effect for group; *F* = 52.937, *p* < 0.001, partial *η*^2^ = 0.524), and stride time variability increased between conditions (significant main effect for condition; *F* = 64.316, *p* < 0.001, partial *η*^2^ = 0.573). For older adults, stride time variability increased significantly across all three conditions (Fig. 1). In contrast, the young adult group only exhibited higher stride time variability during the visually cued gait condition (Fig. 1; significant group × condition interaction effect; *F* = 7.507, *p* = 0.002, partial *η*^2^ = 0.135). Individual data across conditions is visualized in the supplemental material (Fig. S1).

### Stride length variability

**Figure 2:**
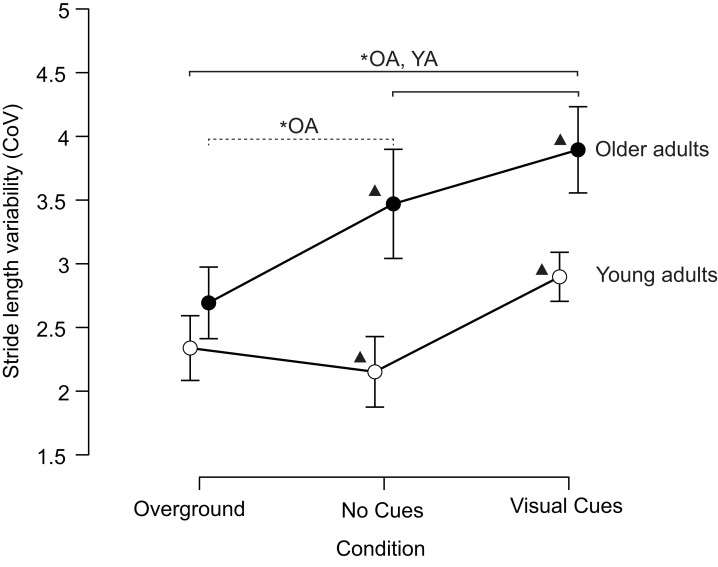
Older adults have higher stride length variability than young adults when walking on a treadmill with and without visual cues. The overground stride length variability was similar between the two groups. CoV is the coefficient of variation. ▴ Indicates statistically significant differences between groups and for the same condition. The solid line indicates statistically significant differences for both groups across different conditions (OA, older adults; YA, young adults). The dashed line indicates statistically significant differences for the older adult group between overground and treadmill without visual cues conditions. *: *p* < 0.05. The error bars indicate the 95% confidence interval.

Older adults walked with higher stride length variability than young adults (significant main effect for group; *F* = 41.565, *p* < 0.001, partial *η*^2^ = 0.464), and stride length variability increased between conditions (significant main effect for condition; *F* = 18.493, *p* < 0.001, partial *η*^2^ = 0.278). The older adult group exhibited an increase in stride length variability from overground to treadmill to visually cued gait. In contrast, the young adult group exhibited an increase in stride length variability from treadmill to visually cued gait only (Fig. 2), indicating a significant group × condition interaction effect (*F* = 5.544, *p* = 0.008, partial *η*^2^ = 0.104). Older adults exhibited higher stride length variability than young adults when walking on the treadmill with and without the visual cues (Fig. 2). Individual data across conditions is visualized in the supplemental material (Fig. S2).

### Local divergence exponent

**Figure 3:**
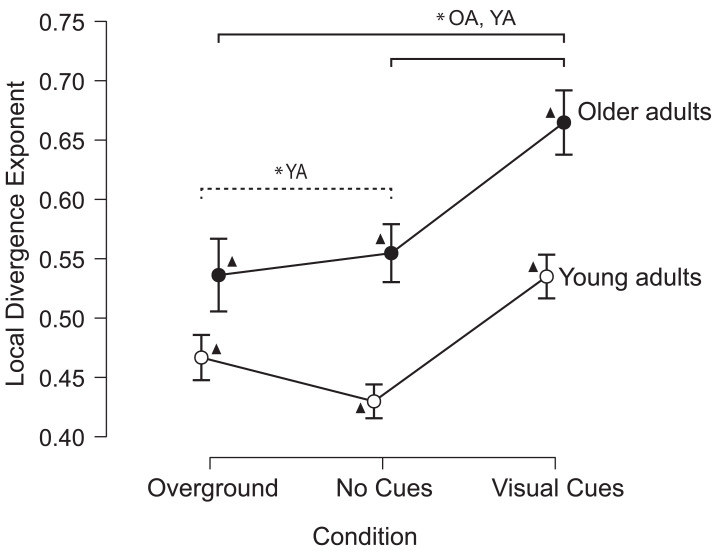
Older adults are less stable than young adults across all walking conditions. Young adults were the most stable when walking on the treadmill. ▴ Indicates statistically significant differences between groups and for the same condition. The solid line indicates statistically significant differences for both groups across different conditions (OA, older adults; YA, young adults). The dashed line indicates statistically significant differences for the young adult group between the overground and treadmill without visual cues conditions. *: *p* < 0.05. The error bars indicate the 95% confidence interval.

Older adults walked with a higher LDE (*i.e.,* less stably) than young adults (significant main effect for the group; *F* = 28.827, *p* < 0.001, partial *η*^2^ = 0.375), and LDE increased between conditions (significant main effect for the condition; *F* = 57.343, *p* < 0.001, partial *η*^2^ = 0.544). Both groups walked with a significantly higher LDE in the visually cued gait condition than in the overground gait condition. Additionally (Fig. 3), young adults walked with a significantly lower LDE (*i.e.,* more stably) in the treadmill gait condition than in the overground gait and the visually cued gait conditions (Fig. 3), indicating a group × condition interaction effect (*F* = 4.524, *p* = 0.013, partial *η*^2^ = 0.086). Older adults walked with a higher LDE than young adults across all three conditions (Fig. 3). Individual data across conditions is visualized in the supplementary material (Fig. S3).

## Discussion

The purpose of our study was to determine whether visually cued gait directly impacts gait variability and dynamic gait stability in young and older adults. Our first hypothesis was partially supported, as only the young adults exhibited higher local dynamic stability (*i.e.,* lower LDE value) during treadmill gait without visual cues compared to overground gait. Contrary to our hypothesis, young adults showed no significant changes in stride time and stride length variability during treadmill gait compared to overground walking. Older adults produced higher stride length and stride time variability during treadmill gait without visual cues compared to overground gait. Our second hypothesis was supported, as both the young and older adult groups walked with the highest stride time and length variability, as well as the highest LDE (least stably) during the visually cued gait condition. Lastly, our third hypothesis was partially supported, such that the older adult group had higher stride time and length variability, as well as a higher LDE than the young adult group during treadmill walking, regardless of visual cues. However, only LDE was significantly higher in older adults compared to young adults during overground gait.

### Gait variability and dynamic instability increase with visual cue walking, regardless of age

Gait variability and local dynamic stability can inform how people respond to environmental changes during gait. When people walk on the street, constant interactions with objects, obstacles, and obstructions can require adjustments that alter gait variability and dynamic stability (Schmitt et al., 2021; Hybart & Ferris, 2023). Importantly, gait variability and stability can be affected by environmental or contextual factors and depend on the method of quantification, as suggested in previous studies (Dingwell et al., 2001; Schmitt et al., 2021; Hybart & Ferris, 2023; Lang et al., 2025). Our study offers further support that overground and treadmill gait have distinct effects on variability and dynamic stability patterns. Specifically, our findings indicate that both stride time and stride length variability are higher for older adults for treadmill gait than for overground gait. These results contrast previous reports that both young and older adults have higher stride time variability during overground gait than for treadmill gait (Row Lazzarini & Kataras, 2016; Bizovska et al., 2018b). Regarding stride length variability, Hybart & Ferris (2023) reported higher variability in young adults during overground gait than during treadmill gait. Additionally, incorporating visual cues during treadmill gait can exacerbate changes in gait performance, potentially due to the increased cognitive load. Terrier (2016) compared auditory and visual cue information during treadmill gait and reported that stride length and stride time variability were the highest with visual cues in young adults. In agreement with this, our results indicate that gait variability increases when young adults walk with visual cues, with the highest stride time and stride length variability during visually cued gait. Ultimately, gait variability changes are present across different gait conditions, and the direction and magnitude of change appear to depend on the surface (*i.e.,* overground *versus* treadmill) and visual input (*i.e.,* no cue *versus* cue).

Changes in gait variability alone may not be enough information to determine whether a person is, in fact, at risk of falling (Toebes et al., 2012; Bruijn et al., 2013; Tamburini et al., 2018). Assessing dynamic gait stability can improve the interpretation of changes in gait variability. Studies that investigated the LDE between overground and treadmill gait suggest that gait stability increases (lower LDE) for young people on a treadmill compared to overground (Dingwell et al., 2001; Buzzi et al., 2003; Terrier & Dériaz, 2011; Bizovska et al., 2018b; Amirpourabasi et al., 2022). In agreement with previous studies, our results indicate that young people walked more stably on the treadmill than overground. This may be a consequence of walking on a treadmill at a constant speed, suppressing low-frequency drift that could affect the LDE value (Dingwell & Cusumano, 2000). In contrast to a previous report (Amirpourabasi et al., 2022), our results indicate that older adults exhibit a higher LDE than young adults for treadmill gait. Moreover, treadmill gait with visual cues further reduces gait stability, a finding that is independent of age. McAndrew, Wilken & Dingwell (2011) found that young adults exhibited lower dynamic gait stability (higher LDE) during perturbed treadmill walking (with low-amplitude visual perturbations) than during unperturbed treadmill gait. Although our protocol did not include a perturbation that may directly influence the vestibular system, our findings suggest that the addition of visual flow information during treadmill gait affects dynamic gait stability in both young and older adults.

Overall, we provide more evidence that highlights how treadmill elicits gait variability and dynamic stability patterns that differ from those of overground gait. When people walk on a treadmill with visual cues, gait variability increases and dynamic gait stability decreases in young and older adults. Consequently, researchers and clinicians should be aware of the effect of walking context and environment when using a treadmill for research and rehabilitation. Moreover, researchers and clinicians may need to consider how age can affect the effectiveness of rehabilitation protocols that use visual feedback, such as augmented reality, virtual reality, or information projected on a screen in front of the participants. Though treadmills are a valuable tool that allows researchers and clinicians to gain fundamental knowledge about gait, it is often considered controlled (Schmitt et al., 2021), whereas daily-life gait is at the other end of the spectrum (*i.e.,* free and uncontrolled). A potential bridge to close the gap between these two contrasting ideas is to develop conditions that emulate human responses to a free-living environment while walking on a treadmill (Schmitt et al., 2021). We propose visually cued gait as an approach to bridge that gap, as it has been shown that vision is fundamental to proper foot placement when walking outside the laboratory (Matthis, Yates & Hayhoe, 2018). Visually cued gait may trigger cortical adaptation that changes gait patterns to meet the requirement of stepping toward the visual target (Matthis, Barton & Fajen, 2017). Thus, visually cued gait on a treadmill may be considered a new context for participants in which they engage the supraspinal system, affecting gait variability and stability, regardless of age.

### Gait variability and local dynamic stability are exacerbated by age

Increases in gait variability can be considered positive or negative outcomes if we consider aging or neurodegenerative conditions (Van Emmerik & Van Wegen, 2000; van Emmerik & van Wegen, 2002; Stergiou, Harbourne & Cavanaugh, 2006). Higher gait variability and lower local dynamic stability in young people are associated with more movement flexibility to meet task and environmental demands (Latash, 2000; Latash, 2012; Latash, Scholz & Schöner, 2002). Some gait variability can be beneficial for a fully functional individual who can react quickly to prevent a fall. As people age, increased gait variability has been associated with a higher risk of falling (Hausdorff, Rios & Edelberg, 2001). We observed that gait variability increases more in older adults than in young adults during gait with visual cues, suggesting that older adults may have greater difficulty processing the additional visual information, exacerbating their gait variability. To confirm this, retrospective or prospective fall quantification is required in combination with gait variability metrics.

Similarly, the LDE is another metric used to predict falls. Higher LDE values in older adults are associated with a higher fall risk (Lockhart & Liu, 2008; Toebes et al., 2012; Ihlen et al., 2016; Tamburini et al., 2018; Bizovska et al., 2018a)**.** Our study suggests that older adults exhibit higher LDE during treadmill gait with and without visual cues than young adults. Thus, gait variability and local dynamic stability appear to be exacerbated by age.

Despite the association between fall risk and both gait variability and LDE, it is difficult to determine whether these outcome measures in isolation can predict fall incidents. Other factors can impact walking performance and increase fall risk. For example, muscle strength, a property associated with muscle function, declines with aging (Goodpaster et al., 2006; Boyer et al., 2023) as well as changes in the nervous system that lead to degradation of sensory information or motor cortex excitability (as reviewed in Boyer et al., 2023). Cognitive load and its link to changes in gait variability and stability is another possible factor that has been explored less (Boyer et al., 2023). Traditionally, cognitive and motor control theories are often considered independent of the habitual or automatic motor tasks (*i.e.,* walking). However, several researchers have proposed that there is an interplay between cognition and motor control for habitual or automatic behavior (Georgopoulos, 2000; Reuter-Lorenz & Cappell, 2008; Gentsch et al., 2016). Reuter-Lorenz & Cappell (2008) demonstrated that older adults may reach a cognitive load ceiling, interfering with their performance on more complex tasks. When young and older adults perform a dual-task, the increased cognitive load leads to higher gait variability in both groups, but with a more pronounced effect in older adults (Decker et al., 2016). Further, increased levels of dementia and cognitive impairments appear to be associated with increased gait variability and fall risk (Amboni, Barone & Hausdorff, 2013). We used visual cues to test whether age is associated with higher gait variability and higher LDE (less stability) as gait gets more complicated. Although we administered the MoCA test to the older adult group to establish each participant’s cognitive baseline, we did not assess cognitive load during any gait condition. Thus, we are unable to determine whether cognitive load directly influences gait variability and dynamic stability in older adults. Nevertheless, the data reported in this study highlight that older adults have higher gait variability and higher LDE than younger adults when walking gets more complicated, which may be associated with cognitive deficits due to aging. Our results support the Compensation-Related Utilization of Neural Circuits Hypothesis (CRUNCH) model (Reuter-Lorenz & Cappell, 2008), which suggests that older adults adopt a cognitive strategy when walking becomes more complicated. The potential interplay between cognitive load, sensory system decline, and muscle strength is not yet fully understood. Future studies should consider implementing formal sensory function and muscle strength assessments as a baseline, as well as assessing cognitive load during walking. This could help parse the interplay among muscle function, central nervous system function, and cognition.

## Limitations and Future Directions

We calculated LDE from 11 bouts of eight strides. Previous work has suggested that the lowest number of bouts to accurately assess LDE is 15 bouts of eight strides (Van Schooten et al., 2014). Some of our participants did not reach 15 bouts of eight strides during their 3 min of overground gait. For participants who completed 15 bouts, we performed a paired *t*-test to determine whether 11 bouts of eight strides had a significant impact on our local dynamic stability outcome measure. We did not find statistical significance (*t* = −0.385; *p* = 0.701, Cohen’s *d* = 0.098), so we included 11 bouts of eight strides to include the overground condition in our analysis. To our knowledge, no study has used fewer than 15 bouts of gait cycles to calculate LDE. Thus, more studies are needed to establish whether 11 bouts of eight strides can contain sufficient information to assess gait stability. Calculating gait stability from 11 bouts of eight strides could help clinicians implement and assess LDE to determine whether their interventions are effective in promoting changes in gait stability. The increased instability in older adults may, in part, be due to greater discomfort with treadmill walking. Future studies could investigate how different levels of discomfort may impact gait stability.

While we assessed gait variability and stability across different conditions, additional information on gait performance could be obtained by quantifying how well participants placed their feet on the targets in the visually cued gait condition. This is part of future research with this paradigm. Furthermore, future studies that incorporate brain imaging and executive function under a similar testing protocol may aid in interpreting the interplay between visual processing, gait variability, and stability across age groups (*i.e.,* young, middle-aged, and older adults). Likewise, more robust cognitive assessments and cognitive-based motor interference tasks would allow more concrete conclusions to be drawn regarding the relationship between cognitive load and gait variability and stability.

Lastly, visually cued gait may be a new task for both young and older adults, which could prompt learning effects in our protocol. While we indicated that the visual cues are displayed on the ground based on participant-specific stepping patterns and told them as much, we cannot exclude the possibility of a learning effect in our protocol, as it was a new environment and context for our participants. Future studies should consider the potential effect of locomotor adaptation with the use of visual cues based on the error-correction principle (Reisman, Bastian & Morton, 2010), which may lead to a learning effect. Considering multiple visits for both groups may shed light on a potential learning effect of visually cued gait in modifying gait variability and stability.

## Conclusions

Exposing people to a gait condition with visual cues affects gait variability and local dynamic stability, regardless of age. In agreement with previous studies, overground and treadmill gait elicit different biomechanical outcomes for both young and older adults. Furthermore, older adults have higher gait variability and lower dynamic stability than younger adults. Gait with visual cues may be more complicated than gait without visual cues, which may be associated with the cortex’s active control of gait and effect on gait variability and stability. Increasing gait complexity in a laboratory setting accentuates differences in gait performance measures between young and older adults. Although it is difficult to directly relate gait variability and dynamic stability to fall predictions, we speculate that older adults may reach their maximum cognitive capacity when exposed to a flow of visual stimuli (*i.e.,* visual cues), thereby increasing gait variability and decreasing local dynamic stability.

## Supplemental Information

10.7717/peerj.21157/supp-1 Supplemental Information 1 Stride time variability was significantly higher for older adults on the treadmill with and without cues Each dot represents a single participant. The half violin illustrates the sample distribution.

10.7717/peerj.21157/supp-2 Supplemental Information 2 Stride length variability was higher in older adults when walking on the treadmill with and without visual cues Each dot represents a single participant. The half violin illustrates the sample distribution.

10.7717/peerj.21157/supp-3 Supplemental Information 3 The local divergence exponent value during visually cued walking was the highest on average among the two groups Each dot represents a single participant. The half violin illustrates the sample distribution.

10.7717/peerj.21157/supp-4 Supplemental Information 4 Percent coefficient of Variation and short-term Lyapunov Exponent values for each participant Each data point is the average percent coefficient of variation between the left and right leg for the stride time and stride length. YA, young adult; OA, Older adult; No cues = treadmill walking without cues; Visual cues, treadmil walking with visual cues. We provide 25 young adults and 25 older adults data (*N* = 50).

10.7717/peerj.21157/supp-5 Supplemental Information 5 STROBE checklist

## Additional Information and Declarations

### Data Availability

The following information was supplied regarding data availability: The raw data is available in the Supplemental File.
