# Supplementary material for: Gait variability and local dynamic stability are exacerbated by age and visual cues
Source: PeerJ. 2026 May 5;14:e21157. doi: 10.7717/peerj.21157 (PMC13155234; doi:10.7717/peerj.21157)
Supplement: Supplemental Information 5 [file peerj-14-21157-s005.docx]

STROBE Statement—checklist of items that should be included in reports of observational studies

|  | Item No. | Recommendation | Page  No. | Relevant text from manuscript |
| --- | --- | --- | --- | --- |
| **Title and abstract** | 1 | (*a*) Indicate the study’s design with a commonly used term in the title or the abstract | 2 | The purpose of this study was to compare gait variability and dynamic gait stability in young and older adults walking overground and on a treadmill with and without visual cues |
|  |  | (*b*) Provide in the abstract an informative and balanced summary of what was done and what was found | 2 | **Methods.** Both groups walked overground, on a treadmill with and without visual cues at their self-selected overground walking speed for 3 minutes. The visual cues were projected onto the treadmill and matched the participant’s self-selected step length. We quantified gait variability as the coefficient of variation for stride time and stride length, and dynamic gait stability with the short-term Lyapunov exponent.  **Results.** Both groups had the highest stride time variability in the visually cued condition. Older adults demonstrated higher gait variability and lower dynamic gait stability than younger adults during treadmill walking with and without visual cues, but not during overground walking. |
| Introduction | | | |  |
| Background/rationale | 2 | Explain the scientific background and rationale for the investigation being reported | 5 | Both gait variability and stability change based on age, visual feedback, and walking environment (i.e., overground versus treadmill) (McAndrew, Wilken & Dingwell, 2011; Wuehr et al., 2013; Francis et al., 2015). However, there is limited evidence across these factors measured in matched experimental conditions, limiting our ability to draw strong conclusions. |
| Objectives | 3 | State specific objectives, including any prespecified hypotheses | 5 | We aimed to quantify gait variability and stability during overground, treadmill, and visually cued treadmill walking in young and older adults. We hypothesized that stride time and length variability would increase, and that dynamic gait stability would decrease (i.e., higher LDE) between overground and treadmill walking. We also hypothesized that stride time and length variability would increase, and dynamic gait stability would decrease during visually cued treadmill walking compared to treadmill walking without visual cues in both young and older adults. Finally, we hypothesized that older adults would walk with higher stride time and stride length variability and lower dynamic gait stability than young adults across all three gait conditions. |
| Methods | | | |  |
| Study design | 4 | Present key elements of study design early in the paper | 6 - 7 |  |
| Setting | 5 | Describe the setting, locations, and relevant dates, including periods of recruitment, exposure, follow-up, and data collection | 6 | The inclusion criteria were that young adults were between 18 and 29 years old, while older adults were 65 years old or older. The exclusion criteria comprised having a chronic musculoskeletal or neurological injury or disease, undergoing surgery or experiencing an injury that would affect gait within the past year, having a cardiovascular disease or arrhythmia, uncorrected vision impairment, or being unable to understand written and spoken English. |
| Participants | 6 | (*a*) *Cohort study*—Give the eligibility criteria, and the sources and methods of selection of participants. Describe methods of follow-up  *Case-control study*—Give the eligibility criteria, and the sources and methods of case ascertainment and control selection. Give the rationale for the choice of cases and controls  *Cross-sectional study*—Give the eligibility criteria, and the sources and methods of selection of participants |  |  |
|  |  | (*b*) *Cohort study*—For matched studies, give matching criteria and number of exposed and unexposed  *Case-control study*—For matched studies, give matching criteria and the number of controls per case | N/A |  |
| Variables | 7 | Clearly define all outcomes, exposures, predictors, potential confounders, and effect modifiers. Give diagnostic criteria, if applicable | 7 | Our primary gait variability outcome measures were the percent coefficient of variation of stride time and of stride length. We used custom MATLAB scripts to calculate the mean stride length and time variability between the left and right legs. We then calculated the group percent coefficient of variation as %CV= ((Standard deviation)/mean)×100.  For gait stability, we used the raw acceleration data from IMUs at the lumbar spine to calculate the LDE using an open-source MATLAB script (Bruijn, 2023) and following previous work (Bruijn et al., 2013; Van Schooten et al., 2014; Mehdizadeh, 2019). Briefly, we time-normalized the data to 100 samples, implemented a time delay of 25 samples (1/4 of the gait cycle), and implemented a 9D space from the three acceleration vectors (XYZ) (Van Schooten et al., 2014). |
| Data sources/ measurement | 8* | For each variable of interest, give sources of data and details of methods of assessment (measurement). Describe comparability of assessment methods if there is more than one group | 7 | Our primary gait variability outcome measures were the percent coefficient of variation of stride time and of stride length. We used custom MATLAB scripts to calculate the mean stride length and time variability between the left and right legs. We then calculated the group percent coefficient of variation as %CV= ((Standard deviation)/mean)×100.  For gait stability, we used the raw acceleration data from IMUs at the lumbar spine to calculate the LDE using an open-source MATLAB script (Bruijn, 2023) and following previous work (Bruijn et al., 2013; Van Schooten et al., 2014; Mehdizadeh, 2019). Briefly, we time-normalized the data to 100 samples, implemented a time delay of 25 samples (1/4 of the gait cycle), and implemented a 9D space from the three acceleration vectors (XYZ) (Van Schooten et al., 2014). The LDE was calculated across all conditions using 11 bouts of 8 strides. Previous work suggests that 15 bouts of 8 strides are sufficient for a valid estimation of the LDE (Van Schooten et al., 2014). However, not all older adults covered 15 bouts of the 17-meter walkway during the overground condition, so we could not extract them. Instead, we used 11 bouts across all conditions to increase the total number of participants in our analysis. For the participants who walked at least 15 lengths, we ran a paired t-test comparing 11 bouts versus 15 bouts of 8 strides and found no difference between the two LDE values (t = -0.385; p = 0.701, Cohen’s d = 0.098). |
| Bias | 9 | Describe any efforts to address potential sources of bias | N/A |  |
| Study size | 10 | Explain how the study size was arrived at | N/A |  |

Continued on next page

| Quantitative variables | 11 | Explain how quantitative variables were handled in the analyses. If applicable, describe which groupings were chosen and why | N/A |  |
| --- | --- | --- | --- | --- |
| Statistical methods | 12 | (*a*) Describe all statistical methods, including those used to control for confounding | 7 – 8 |  |
|  |  | (*b*) Describe any methods used to examine subgroups and interactions |  |  |
|  |  | © Explain how missing data were addressed | N/A |  |
|  |  | (*d*) *Cohort study*—If applicable, explain how loss to follow-up was addressed  *Case-control study*—If applicable, explain how matching of cases and controls was addressed  *Cross-sectional study*—If applicable, describe analytical methods taking account of sampling strategy | N/A |  |
|  |  | © Describe any sensitivity analyses | N/A |  |
| Results | | | | |
| Participants | 13* | (a) Report numbers of individuals at each stage of study—eg numbers potentially eligible, examined for eligibility, confirmed eligible, included in the study, completing follow-up, and analysed | 6 |  |
|  |  | (b) Give reasons for non-participation at each stage | 6 | Twenty-five young adults (mean [SD] = 22 [4] years old; females = 15) and twenty-five older adults (mean [SD] = 70 [4] years old; females = 12) participated in this study. Written informed consent was obtained from each participant before the study. All procedures were approved by the Institutional Review Board (protocol #3501) at the University of Massachusetts Amherst. The inclusion criteria were that young adults were between 18 and 29 years old, while older adults were 65 years old or older. The exclusion criteria comprised having a chronic musculoskeletal or neurological injury or disease, undergoing surgery or experiencing an injury that would affect gait within the past year, having a cardiovascular disease or arrhythmia, uncorrected vision impairment, or being unable to understand written and spoken English. |
|  |  | © Consider use of a flow diagram | N/A |  |
| Descriptive data | 14* | (a) Give characteristics of study participants (eg demographic, clinical, social) and information on exposures and potential confounders | N/A |  |
|  |  | (b) Indicate number of participants with missing data for each variable of interest | N/A |  |
|  |  | © *Cohort study*—Summarise follow-up time (eg, average and total amount) | N/A |  |
| Outcome data | 15* | *Cohort study*—Report numbers of outcome events or summary measures over time |  |  |
|  |  | *Case-control study—*Report numbers in each exposure category, or summary measures of exposure |  |  |
|  |  | *Cross-sectional study—*Report numbers of outcome events or summary measures | 10 - 12 |  |
| Main results | 16 | (*a*) Give unadjusted estimates and, if applicable, confounder-adjusted estimates and their precision (eg, 95% confidence interval). Make clear which confounders were adjusted for and why they were included | N/A |  |
|  |  | (*b*) Report category boundaries when continuous variables were categorized | N/A |  |
|  |  | (*c*) If relevant, consider translating estimates of relative risk into absolute risk for a meaningful time period | N/A |  |

Continued on next page

| Other analyses | 17 | Report other analyses done—eg analyses of subgroups and interactions, and sensitivity analyses | 8 | We analyzed all three outcome measures using a 2 × 3 mixed-effect ANOVA (age group × walking condition). A post-hoc test with the Bonferroni adjustment was performed when statistical significance was found for the group × condition interaction. Additionally, a Greenhouse-Geisser correction was applied to the stride time and stride length variability, as sphericity of these two variables was violated. We then performed Bonferroni-adjusted paired t-tests to evaluate statistical significance within each group and across all three conditions. We also performed Bonferroni-adjusted independent t-tests to assess statistical significance within a condition and between the young and older adults. A Mann-Whitney U test was used because the data were not normally distributed for the independent t-test. |
| --- | --- | --- | --- | --- |
| Discussion | | | | |
| Key results | 18 | Summarise key results with reference to study objectives | 10 | The purpose of our study was to determine whether visually cued walking directly impacts gait variability and stability in young and older adults. Our first hypothesis was partially supported, as only the older adults’ group had a higher stride time and length variability and higher LDE from overground to treadmill walking without visual cues. Next, our second hypothesis was supported, as both the young and older adult groups exhibited the highest stride time and length variability, along with the highest LDE (least stable) during the visually cued walking condition. Lastly, our third hypothesis was partially supported, showing that the older adult group had higher stride time and length variability, as well as a higher LDE than the young adult group during treadmill walking, regardless of visual cues. For overground walking, gait variability measures were comparable between groups, but the LDE was elevated in the older adult group. |
| Limitations | 19 | Discuss limitations of the study, taking into account sources of potential bias or imprecision. Discuss both direction and magnitude of any potential bias | 14 | The calculation of the LDE was done with 11 bouts of 8 strides. Previous work has suggested that the lowest number of bouts for LDE is 15 bouts of 8 strides (Van Schooten et al., 2014). Some of our participants did not reach 15 bouts of 8 strides in the overground condition. We performed a paired t-test to determine if 11 bouts of 8 strides had a major impact on our local dynamic stability outcome measure. We did not find statistical significance (t = -0.385; p = 0.701, Cohen’s d = 0.098), so we included 11 bouts of 8 strides to include the overground condition in our analysis. Also, we argue that increased gait variability and local divergence exponent in older adults may be associated with cognitive impairments. Although we only assess baseline cognitive level and not cognitive load in any walking condition, evidence from previous studies (Reuter-Lorenz & Cappell, 2008; Decker et al., 2016) suggests that our observations may indicate that older adults are affected by a cognitive capacity ceiling, which may be manifested through higher gait variability and lower gait stability. |
| Interpretation | 20 | Give a cautious overall interpretation of results considering objectives, limitations, multiplicity of analyses, results from similar studies, and other relevant evidence | 13 | Despite the association between gait variability or LDE and fall risk, it is difficult to determine whether these outcome measures can predict fall incidents. Other factors can impact walking performance and increase fall risk. A possible factor is cognitive load. Reuter-Lorenz and Cappell (2008) have demonstrated that older adults may reach a cognitive load ceiling, interfering with how people respond to a more complex task (Reuter-Lorenz & Cappell, 2008). When young and older adults perform a dual-task, the increased cognitive load causes a higher level of gait variability in both groups, but with a more pronounced effect in older adults (Decker et al., 2016). Further, increased levels of dementia and cognitive impairments have been associated with increased gait variability and fall risk (Amboni, Barone & Hausdorff, 2013). We intended to test whether age is associated with higher gait variability and higher LDE (less stable) when walking gets more complicated, using a flow of visual information. Although we performed the MoCA test in the older adult group to identify each participant’s cognitive baseline, we did not assess cognitive load during any walking condition. Thus, we are unable to determine whether cognitive load directly influences gait variability and stability in older adults. Nevertheless, the data reported in this study highlight that older adults have higher gait variability and higher LDE than younger adults when walking gets more complicated, which may be associated with cognitive impairment because of aging. |
| Generalisability | 21 | Discuss the generalisability (external validity) of the study results | N/A |  |
| Other information | |  | | |
| Funding | 22 | Give the source of funding and the role of the funders for the present study and, if applicable, for the original study on which the present article is based | 15 | We thank Zachary Barrons and Ella Simonson for their assistance with data collections, and Erinne Hau and Andrew Lef for their assistance with data processing. We thank Sina Medizadeh, Kim van Schooten, and Sjoerd Bruijn for sharing their insights and for sharing open-source scripts to calculate the local divergence exponent. We used Grammarly to identify grammatical and syntax errors. This enabled a more thorough revision by concentrating on the content of the manuscript with minimal effort on grammatical and syntax issues. |

*Give information separately for cases and controls in case-control studies and, if applicable, for exposed and unexposed groups in cohort and cross-sectional studies.

**Note:** An Explanation and Elaboration article discusses each checklist item and gives methodological background and published examples of transparent reporting. The STROBE checklist is best used in conjunction with this article (freely available on the Web sites of PLoS Medicine at http://www.plosmedicine.org/, Annals of Internal Medicine at http://www.annals.org/, and Epidemiology at http://www.epidem.com/). Information on the STROBE Initiative is available at www.strobe-statement.org.
